# Coordination of Metabolism and Virulence Factors Expression of Extraintestinal Pathogenic *Escherichia coli* Purified from Blood Cultures of Patients with Sepsis * [Fn FN2]

**DOI:** 10.1074/mcp.M116.060582

**Published:** 2016-06-30

**Authors:** Veronika Kuchařová Pettersen, Knut Anders Mosevoll, Paul Christoffer Lindemann, Harald G. Wiker

**Affiliations:** From the ‡The Gade Research Group for Infection and Immunity, Department of Clinical Science, University of Bergen, N-5021 Bergen, Norway;; §Department of Clinical Science; University of Bergen, N-5021 Bergen, Norway;; ¶Department of Microbiology; Haukeland University Hospital, N-5021 Bergen, Norway

## Abstract

One of the trademarks of extraintestinal pathogenic *Escherichia coli* is adaptation of metabolism and basic physiology to diverse host sites. However, little is known how this common human pathogen adapts to permit survival and growth in blood. We used label-free quantitative proteomics to characterize five *E. coli* strains purified from clinical blood cultures associated with sepsis and urinary tract infections. Further comparison of proteome profiles of the clinical strains and a reference uropathogenic *E. coli* strain 536 cultivated in blood culture and on two different solid media distinguished cellular features altered in response to the pathogenically relevant condition. The analysis covered nearly 60% of the strains predicted proteomes, and included quantitative description based on label-free intensity scores for 90% of the detected proteins. Statistical comparison of anaerobic and aerobic blood cultures revealed 32 differentially expressed proteins (1.5% of the shared proteins), mostly associated with acquisition and utilization of metal ions critical for anaerobic or aerobic respiration. Analysis of variance identified significantly altered amounts of 47 proteins shared by the strains (2.7%), including proteins involved in vitamin B6 metabolism and virulence. Although the proteomes derived from blood cultures were fairly similar for the investigated strains, quantitative proteomic comparison to the growth on solid media identified 200 proteins with substantially changed levels (11% of the shared proteins). Blood culture was characterized by up-regulation of anaerobic fermentative metabolism and multiple virulence traits, including cell motility and iron acquisition. In a response to the growth on solid media there were increased levels of proteins functional in aerobic respiration, catabolism of medium-specific carbon sources and protection against oxidative and osmotic stresses. These results demonstrate on the expressed proteome level that expression of extraintestinal virulence factors and overall cellular metabolism closely reflects specific growth conditions. Data are available via ProteomeXchange with identifier PXD002912.

*Escherichia coli* is a colonizer of the lower intestine of humans and other warm-blooded vertebrates. A subgroup of these mostly harmless bacteria, termed extraintestinal pathogenic *E. coli* (ExPEC) ^1^, has a capacity to invade and colonize the urinary tract, the bloodstream, and cerebrospinal fluid of the hosts. The ability of ExPEC to persist in different host sites has an origin in the dynamic nature of the *E. coli* genome: various *E. coli* subspecies can share as little as 20% of essential genes, and the rest of the genome contains strain specific DNA, also called a flexible gene pool (1, 2). Comparative genomic studies of commensal and pathogenic *E. coli* strains suggest that it is the specific composition of this flexible genome, and particularly genetic material acquired horizontally via transduction, conjugation and transformation, which determines the ability of *E. coli* to cause certain diseases and to be recognized as a specific pathotype (3–5). Pathogenic islands acquired by horizontal gene transfer and containing genes directly linked to ExPEC virulence (6), together with an exceptionally high level of recombination of ExPEC isolates when compared with commensal strains (7), further corroborate that the plasticity of the *E. coli* genome is one of the bases of ExPEC pathogenicity. Nevertheless, the interpretation of the genome content, *i.e.* the presence or absence of specific genes, is alone not sufficient for drawing a detailed picture of bacterial pathogenesis. In this context, cell-wide descriptions of protein quantitative levels, which point to the level of gene regulation, are essential in order to expand strategies for treatment and prevention of ExPEC infections.

Currently, there is not an effective vaccine to prevent ExPEC infections (8), the most serious of which is septicemia, a condition with a high mortality rate. The difficulties in finding an agent for prevention of ExPEC-mediated diseases are partly caused by one striking aspect of ExPEC pathogenesis: lack of a single dominant virulence factor or a common set of virulence determinants shared by the ExPEC strains, and not present in the commensal or intestinal pathogenic *E. coli* (4, 9, 10). Extraintestinal virulence is a multigenic process including genes encoding transcriptional regulators (11), iron and heme receptors (12), fimbrial adhesins (13), toxins (14), and proteins functional in cell motility (15, 16) and biosynthesis of lipopolysaccharides and polysaccharide capsules (17). Moreover, it appears that many of the factors responsible for virulence are primarily associated with gut colonization rather than being typical virulence factors directly involved in infection (18, 19). Over the last few years, evidence has been accumulating that general metabolism has paramount importance in ExPEC virulence (20, 21). Similarly to pathogenic islands carrying virulence genes, metabolic pathways encoded by horizontally acquired genomic elements can provide an advantage and allow adaptation to niches unable to be colonized by commensal *E. coli* strains (6).

Shotgun proteome analysis based on LC-MS/MS is currently a well-established method for identification of thousands of cellular proteins. Protein quantification has traditionally been based on a labeling strategy; however, label-free quantification (LFQ) is simple, applicable to any kind of sample, relatively low cost and scales well when it comes to the number of samples (22). There are multiple benefits of cell-wide protein quantification for clinical microbiology and vaccine development: different pathogenic strains of the same species often display virulence characteristics that necessitate specific treatment, and in a similar manner, extraintestinal pathogens such as uropathogenic *E. coli* (UPEC) cannot be clearly discriminated from non-pathogenic *E. coli* by molecular epidemiological approaches (9). The LFQ proteomic concept therefore makes an ideal framework for a MS-based method that confidently distinguishes between different pathogenic strains as well as between related commensal and pathogenic bacteria (23, 24).

The primary aim of this study was to investigate how *E. coli* strains associated with sepsis and urinary tract infections respond to an *in vitro* culture of human blood at the proteome level. Blood cultures are a crucial part of the evaluation of patients with suspected sepsis, and provide a sensitive means for recovering microorganisms from blood. By using comparative LFQ analysis we determined that multiple proteins functional in anaerobic fermentative metabolism, amino acid metabolism and biosynthesis of specific cofactors, together with known ExPEC virulence traits linked to cell motility and iron acquisition, were expressed in significantly higher amounts in blood culture than during aerobic growth on either nutrient-rich or -limited solid media.

## EXPERIMENTAL PROCEDURES

### 

#### 

##### Ethical Statement

The bacterial samples used in this research originated from blood samples collected for routine microbiological tests, which are made on a regular basis; therefore, no additional procedures were carried out on the patients. Samples were analyzed after written informed consent from the patients. A healthy volunteer donated blood sample, which was used for blood culture of UPEC 536. The regional ethical committee approved the study (REK-vest nr 2013–102).

##### Blood Cultures and Microbiological Characterization of Clinical Isolates

The blood specimens (10 ml per blood culture) were collected from adult patients with suspected sepsis, which were hospitalized at the Haukeland University Hospital (HUH), Bergen, Norway, in the period from November 2014 to May 2015. The blood cultures were drawn for clinical purposes prior to any antibiotic treatment, and detection/identification of bacterial species was conducted at the Department of Microbiology, HUH, according to the accepted clinical standard. Automated blood culture system BacT/ALERT 3D (bioMérieux, Inc., Durham, NC) was used for microbial growth detection. Blood culture broths (BacT/ALERT® FA Plus with aerobic conditions and BacT/ALERT® FN Plus with anaerobic conditions; bioMérieux Inc.) identified as positive by the BacT/ALERT 3D instrument were analyzed using microflex LT, a MALDI-TOF MS instrument (Bruker Daltonics GmbH, Bremen, Germany) for direct species identification. The time for development of positive blood cultures varied between 8–22 h. Antimicrobial susceptibility testing by disk diffusion (Oxoid Lmt., Hampshire, UK) was done according to guidelines and clinical breakpoints established by the European Committee on Antimicrobial Susceptibility Testing (EUCAST). Antibiotic disks used for the tests included: ampicillin, amoxicillin-clavulanate, piperacillin-tazobactam, cefuroxime, ceftazidime, cefotaxime, meropenem, gentamicin, ciprofloxacin, and trimethoprim/sulfamethoxazole. Isolate G5m was in addition tested against cefepime and tigecycline. Reference strain 536 was used for inoculation of anaerobic/aerobic blood culture pair injected with blood donated from a volunteer. These cultures were processed identically as the clinical blood cultures, after being flagged positive for microbial growth by the BacT/ALERT 3D instrument.

##### Isolation of Bacteria from Blood Culture Bottles and Cell Lysis

Broth from each blood culture was first separated from adsorbent polymeric beads, which are used for antimicrobial neutralization, by a sterile sieve. Next, 30 ml of the broth was mixed in a ratio 2:1 with lysis buffer (0.6% polyoxyethylene 10-oleoyl ether [Brij 97] in 0.4 m [3-(cyclohexylamino)-1-propane sulfonic acid] [CAPS]; filtered through a 0.2-μm-pore-size filter, pH 11.7), vortexed for 5s, and allowed to incubate for 5 min at room temperature. The bacterial cells were separated from lysed blood cells by centrifugation at 4495 × *g* for 20 min. Resulting supernatant was discarded and the bacterial cells were washed 3x with a washing buffer (20 mm Na_2_H_2_PO_4_ 2H_2_O, 0.05% Brij 97, and 0.45% NaCl; filtered through a 0.2-μm-pore-size filter, pH 7.2) and 2x with Tris-buffered saline (50 mm Tris, 150 mm NaCl), under same centrifugation conditions as described above. Isolated bacterial cells were frozen at −70 °C until further processing. The cell pellet was then resuspended in an extraction buffer (2.5% SDS, 10 mm Tris/HCl, pH 8.0) and transfered to a lysing matrix A tube (MP Biomedicals, Santa Ana, CA). The cell lysis was performed mechanically by bead beating in a FastPrep FP120 Cell Disrupter (Qbiogene Inc. Carlsbad, CA) for 60 s at maximum speed (6.5 m/s). The cell extracts were cooled on ice for 5 min and centrifuged at 20,000 × *g* for 30 min at 4 °C. The protein content of the supernatant was quantified by using the Direct Detect® method (EMD Millipore, Billerica, MA).

##### Detection of Genetic Determinants by PCR

Phylogrouping of the clinical isolates was done as previously described (25) by the quadriplex PCR method that targets three phylogenetic group marker genes (*chuA*, *yjaA,* and *arpA*) and a DNA fragment TspE4.C2, which is as part of a putative lipase esterase gene. The isolates were allocated to different clonal lineages by multilocus sequence typing (MLST) (26), which is based on sequencing internal fragments of seven housekeeping genes (*adk*, *fumC*, *gyrB*, *icd*, *mdh*, *purA*, and *recA*). The alleles and sequence type (ST) were assigned in accordance with the *E. coli* MLST databases at the University of Warwick. Positive and negative controls were included in all PCR assays.

##### Harvesting Bacterial Cells from Solid Media

In two independent biological replicates, UPEC 536 was streaked either onto fresh blood or lactose agar plates, and cultured at 37 °C for 16 h. Several colonies were then resuspended in Lysogeny broth, and an aliquot of this suspension was pipetted onto new agar plate and evenly distributed to make a lawn. After 16h incubation at 37 °C (stationary growth phase), bacterial cells were scraped from the agar surface and resuspended in Tris-Buffered Saline (TBS). The cells were pelleted by centrifugation at 1500 × *g* for 10 min and then again resuspended in TBS. The washing step was repeated two times. Resuspension of the cell pellet in SDS extraction buffer and cell lysis was performed as described in the section on isolation of bacteria from blood cultures.

##### Filter-aided Protein Digestion

The whole cell lysates were processed according to the Multiple Enzymes for sample Digestion- Filter-Aided Sample Preparation (MED-FASP) protocol using LysC and trypsin (27). The resulting peptide mixtures were first desalted by using in-house made RP-C18 STAGE tips (28), then lyophilized at 30 °C in a vacuum concentrator (Concentrator Plus®, Eppendorf, Hamburg, Germany) and stored in −70 °C until further analysis. Prior to the LC-MS/MS the peptide mixtures were resuspended in 0.1% formic acid and 2% ACN.

##### LC-MS/MS

The MS/MS analysis was carried out at the Proteomics Unit at the University of Bergen on an Ultimate 3000 RSLC system (Thermo Scientific, Waltham, MA) connected to a LTQ Orbitrap mass spectrometer (Thermo Scientific) equipped with a nanoelectrospray ion source. Briefly, 0.5–1 μg protein was loaded onto a preconcentration column (Acclaim PepMap 100, 2 cm × 75 μm ID nanoViper column, packed with 3 μm C18 beads) at a flow rate of 5 μl/min for 5 min using an isocratic flow of 0.1% TFA (v/v) with 2% ACN (v/v). Peptides were separated during a biphasic ACN gradient from two nanoflow UPLC pumps (flow rate of 270 nl/min) on the analytical column (Acclaim PepMap 100, 50 cm × 75 μm ID nanoViper column, packed with 3 μm C18 beads). Solvent A and B was 0.1% TFA acid (v/v) in water or ACN, respectively. Separated peptides were sprayed directly into the MS instrument during a 195 min LC run with the following gradient composition: 0–5 min 5% B, 5–6 min 5–7% B, 6–135 min 7–32% B, 135–145 min 32—% B, 145–150 min 40–90% B. Elution of very hydrophobic peptides and conditioning of the column was performed by isocratic elution with 90% B (150–170 min) and 5% B (175–195 min), respectively. Desolvation and charge production were accomplished by a nanospray Flex ion source.

The mass spectrometer was operated in data-dependent-acquisition mode to automatically switch between Orbitrap-MS and LTQ-MS/MS acquisition. Survey of full-scan MS spectra (from *m*/*z* 300 to 2000) were acquired in the Orbitrap with resolution of *r* = 240,000 at *m*/*z* 400 (after accumulation to a target of 1,000,000 charges in the LTQ). The method used allowed sequential isolation of the most intense ions (up to 12, depending on signal intensity) for fragmentation on the linear ion trap using collisionally induced dissociation at a target value of 10,000 charges. Target ions already selected for MS/MS were dynamically excluded for 40s. General MS conditions were as follows: electrospray voltage, 1.8 kV; no sheath; and auxiliary gas flow. Ion selection threshold was 3000 counts for MS/MS. and an activation Q-value of 0.25 and activation time of 10 ms was also applied for MS/MS.

##### MS/MS Data Analysis

All MS raw data files were processed together in MaxQuant (version 1.5.3.28) (29). Andromeda search engine integrated in the MaxQuant framework (30) performed the spectra search against the following databases: reviewed *E. coli* proteins from the Swiss-Prot section of the UniProtKB database (28,659 entries, downloaded on the 7th October 2015), and predicted proteomes of three genome-sequenced UPEC strains downloaded from NCBI (October 2015): CTF073 (NCBI Reference Sequence NC_004431.1; 5,364 entries for chromosome), 536 (NC_008253.1; 4,619 entries for chromosome) and EC958 (NZ_HG941718.1, NZ_HG941719.1 and NZ_HG941720.1; 4957 and 144 entries for chromosome and plasmids, respectively). UPEC strains CTF073 and EC958 represented the same sequence types as four of the clinical ExPEC isolates (ST73 and ST131). Enzyme specificity was defined in group-specific parameters as either to trypsin or LysC, allowing N-terminal cleavage to proline, and as many as two missed cleavages were allowed. Data for LysC and tryptic fractions originating from the same replicate were combined in MaxQuant. Standard settings were used for MaxQuant searches, except that lysine acetylation and glutamate/glutamine conversion to pyro-glutamate were set as variable modifications in addition to N-terminal acetylation and methionine oxidation. Carbamidomethylation of cysteines was set as a fixed modification. The initial allowed mass deviation of the precursor ion was as high as 20 ppm, and the allowed value for the fragment mass was as high as 0.5 Da. The “match between runs” option was enabled to match identifications across samples. The maximum false discovery rates (FDR) at peptide and protein levels were specified as 0.01. Normalized spectral proteins intensities (LFQ intensity) were derived by the MaxLFQ algorithms (31). For 112 proteins identified by single peptide, detailed information about the MS/MS spectrum, peptide sequence, and precursor *m*/*z* is provided (supplemental Table S1).

We analyzed MaxQuant output data with the Perseus module (http://www.perseus-framework.org, version 1.5.1.6). Protein functional analysis was performed using the DAVID (32) and STRING (33) tools, together with the EcoCyte (34), and UniprotKB databases (35). Sequence alignments of protein isoforms were performed with protein-protein BLAST (NCBI) and Clustal Omega (EMBL-EBI). Cellular localization prediction was made by PSORTb version 3.0.2 (36). MS data were deposited in the PRIDE Proteomics IDEntifications database (37) under accession number PXD002912.

##### Experimental Design and Statistical Rationale

Samples for LC-MS/MS analysis included two or four blood cultures from a patient, two blood cultures from a healthy volunteer, and two biological replicates for samples derived from agar plates. At least two technical replicates of each biological sample were included, and all samples were analyzed in a random order. A control mixed sample was prepared by combining equal volumes of processed peptide mixtures, which were derived from anaerobic and aerobic blood cultures of five strains (C4n, D4n, E4n, G5m, and 536). For qualitative proteome descriptions proteins identified in at least two replicates in blood culture (including anaerobic and aerobic condition), on blood agar or lactose agar were considered valid. For the LFQ analysis, only proteins identified in at least two replicates of a specific culturing condition, with the distinction of anaerobic and aerobic blood culture, were considered valid. Proteins with significant differential abundance were identified by statistical analysis based either on analysis of variance or on two-sided *t* test, which were performed on proteins log_2_ transformed LFQ values. In both cases FDR was kept at 1%. Technical replicates were grouped during FDR calculation that was performed by a permutation-based procedure with 250 randomizations, similar to the procedure applied to FDR calculation for differential expression analysis (38). Principal component analysis (PCA) was done on logarithmized LFQ values in Perseus (FDR = 1%), and details of the PCA implementation were previously described (39).

## RESULTS

### 

#### 

##### ExPEC solates from Clinical Blood Cultures Associated with Sepsis

Patient blood is one of the most important specimens in clinical microbiology and it is collected in commercial blood culture bottles containing growth-promoting additives, which are subsequently incubated in an automated microbial detection system. At Haukeland University Hospital (HUH) bacterial isolates are routinely characterized by MALDI-TOF MS. We took an advantage of the fact that when a blood culture is flagged as positive for bacterial growth, it contains enough bacterial cells for the MS analysis, and at the same time the bacteria have been cultured in media containing up to 20% of patient blood. Once the Department of Microbiology, HUH, confirmed a positive blood culture from a patient included in the study, we purified bacterial cells at stationary growth phase, ∼24 to 48 h after the blood sample was collected from the patient. Depending on the availability, two or four blood cultures from one patient were obtained, and these included either anaerobic, aerobic or both conditions. In total, we acquired clinical blood cultures from 5 patients with confirmed *E. coli* sepsis (Table I).

**Table I:** E. coli strains used in this study. Abbreviations: UTI–Urinary Tract Infection; Trim/Sulfa - Trimethoprim/Sulfamethoxazole; N - Anaerobic; O - Aerobic, M - Male, F - Female

*E. coli* strain	Clinical characteristics (patient sex and age)	MALDI-TOF identification		Resistance to antibiotics	Sequence type (ST), Phylogenetic group	Nr. of blood cultures
		Blood culture	Urine			
C4n	UTI with sepsis (M76)	*E. coli*	*E. coli*	Ciprofloxacin, Trim/Sulfa	ST131, B2	1 N, 1 O
D4n	UTI with sepsis (F84)	*E. coli*	Mixed sample*^a^*	-	ST73, B2	2 N
E4n	UTI with sepsis (M78)	*E. coli*	*E. coli*	Ciprofloxacin, Trim/Sulfa	ST131, B2	2 O
F4d	UTI with sepsis (F90)	*E. coli*	Mixed sample*^a^*	-	ST73, B2	2 N, 2 O
G5m	Immunosuppression with sepsis (M65)	*E. coli*	*E. coli*	-	ST372, B2	2 N, 2 O
UPEC 536	Blood from healthy volunteer (M55)	*-*	*-*	-	ST92, B2	1 N, 1 O

*^a^* The samples were not investigated by MALDI-TOF analysis because of the mixed nature. However, they most likely contained *E. coli* bacteria in a significant amount.

Phylogenetic analysis by the Clermont typing method (25) assigned the five clinical strains to the phylogroup B2 (supplemental Fig. S1). The genetic background of the strains was further characterized by multilocus sequence typing (Table I). The determined sequence types (ST) were previously associated with urinary tract infections (UTI) (40), which was consistent with the fact that the patients also suffered from UTI. In addition, four of the clinical strains belong to two of the most common lineages of UPEC clones: globally disseminated *E. coli* ST131 and ST73. ST131 isolates displayed resistance against ciprofloxacin and trimethoprim/sulfamethoxazole. Lastly, we included a reference UPEC strain 536, which was used for inoculation of blood cultures supplemented with blood from a healthy person.

##### Deep Coverage of ExPEC Proteome by High-resolution Mass Spectrometry

**Fig. 1.**
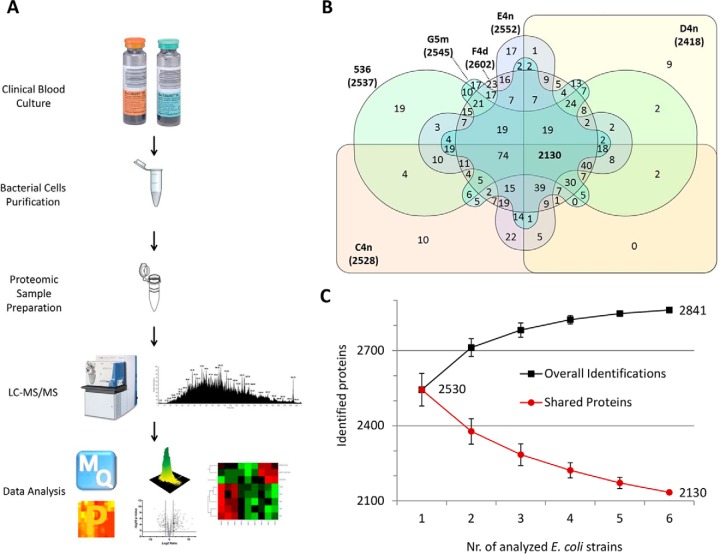
**Detection of ExPEC proteome in blood culture.** *A*, The experimental workflow included the purification of *E. coli* cells from clinical blood cultures, processing of cell extracts, preparation of peptide mixtures for LC-MS/MS, and data analysis by MaxQuant (MQ) and Perseus (P) software. *B*, The Venn diagram displays numbers of identified proteins for 5 clinical *E. coli* isolates and UPEC 536, and distribution of protein identifications between the strains. *C*, Including several ExPEC strains in the proteomic analysis had an additive effect on the number of totally identified proteins. The number of proteins shared by the strains on the other hand decreased with each added strain.

The goal of this study was to achieve a substantial coverage of ExPEC proteome produced under *in vitro* clinical condition of a blood culture. Accordingly, we applied a consecutive two-step protein digestion protocol (27) on whole cell lysates prepared from the five clinical *E. coli* strains and the UPEC strain 536, after purification of the respective *E. coli* cells from the blood culture bottles (Fig. 1*A*). LC-MS/MS analysis of the resulting peptide mixtures generated ∼4.4 million spectra. The acquired raw MS data files were then analyzed by an automated computational pipeline in the MaxQuant environment (41), which matched the spectral files to ∼37 thousand unique peptides (supplemental Table S2). We identified altogether 2841 proteins in the blood culture condition (supplemental Table S3), with an average of 2530 proteins per strain. Approximately 4% of the proteins were identified by only one unique peptide (supplemental Table S1) and therefore represented less confident identifications. About 75% of the detected proteins were common for all strains, and only some proteins were identified in one strain only (Fig. 1*B*). These proteins were often described by putative functions and marked by a low number of observations in the MS, suggesting a low cellular abundance close to the limit of MS detection. This is an indication that they are likely to be present in one or more of the other strains, but in amounts below the detection limit.

Similarly to comparative genomic studies (1, 2), analysis of several ExPEC strains led to an increase in the total number of identified proteins, while the number of proteins shared by the strains gradually decreased with the number of analyzed strains (Fig. 1*C*). Comparison of functional annotation and cellular localization of the detected proteins among the strains showed very similar coverage of proteins within the main functional classes (supplemental Table S4 and S5). Additional evidence that our protein data set covered a significant portion of each strain's expressed proteome was identification of 27 alternative protein isoforms (supplemental Table S6).

##### Quantitative Proteome Profiles of Six ExPEC Strains

Protein quantification permits characterization of those proteins that are differentially expressed among different strains or in different growth conditions. Here we utilized an optional parameter in MaxQuant, which is label-free quantification (LFQ) (31). We obtained LFQ intensities for 90% of the identified proteins (2549). Their quantitative levels, with reference to LFQ intensity scores, covered a 5-log_10_ dynamic range (supplemental Table S7) and correlations between replicates represented as Pearson correlation coefficient R varied between 0.95–0.99 (supplemental Table S8). The LFQ intensities distributions (*i.e.* quantitative protein profiles) were very similar for the six investigated strains (supplemental Fig. S2*A*), nevertheless, PCA suggested differences in proteins quantitative levels between anaerobic and aerobic blood culture conditions, as well as for individual strains (supplemental Fig. S2*B*). Specifically, the PCA score plot showed visible separation of samples originating from the two blood culture conditions of the same strains. At the same time, samples from different strains but from the same growth condition grouped more closely together.

##### Levels of Many ExPEC Proteins Vary with Presence/Absence of Oxygen in Blood Culture

A routine blood culture for adult patients consists of paired aerobic and anaerobic bottles, and we received one or two bottles of each culturing condition for three patients. The reference strain 536 was also cultured in both blood culture types. Therefore, we first determined if the blood culture atmosphere of N_2_ and CO_2_ either with or without O_2_ had any significant effect on the ExPEC protein profiles. The most apparent result was a higher number of protein identifications under aerobic conditions for three out of four strains (supplemental Table S9). When considering all strains, 40 and 129 proteins were detected exclusively under anaerobic or aerobic conditions, respectively (supplemental Table S10). A majority of these proteins had a low number of peptide identifications from MS/MS data, implicating a low cellular abundance at the limit of detection. Therefore, we do not exclude their expression in the other condition, however; we would expect to see these proteins in reduced amounts. Most of the proteins were membrane-associated transport proteins (around 30% in both conditions), and multiple proteins of oxidation/reduction and cofactor-dependent metabolic processes were detected under aerobic condition.

**Fig. 2.**
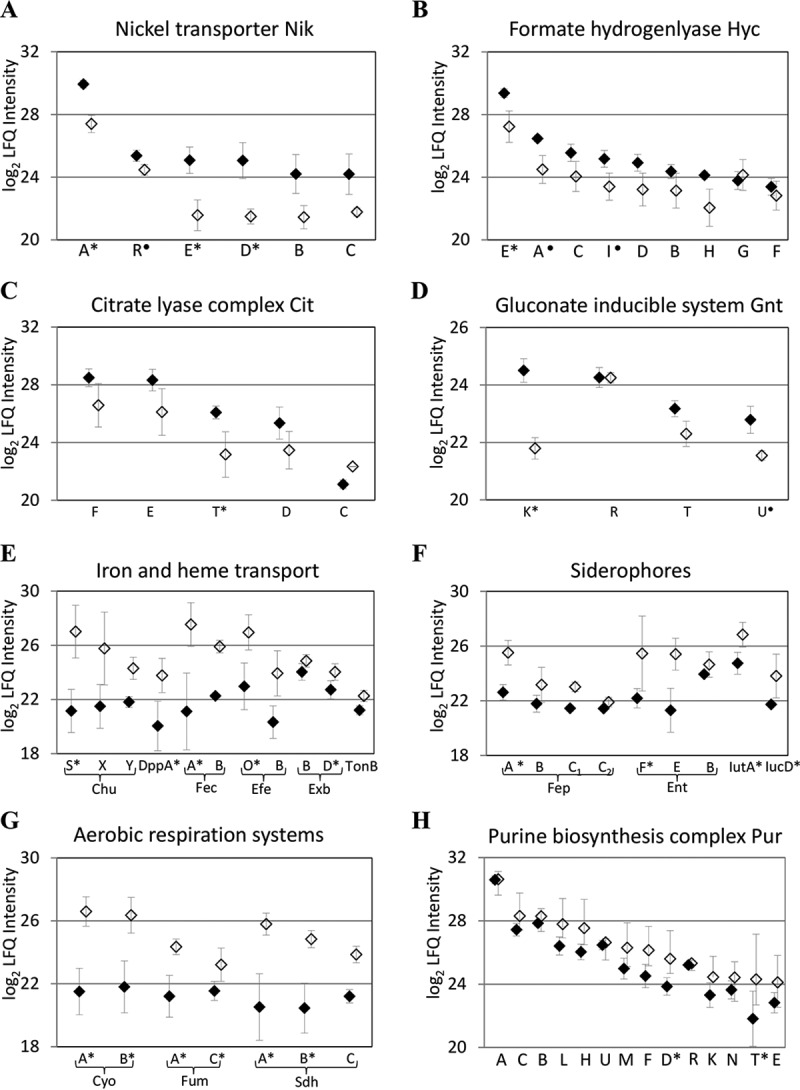
**Quantitative profiles of differentially expressed ExPEC protein complexes under anaerobic and aerobic blood culture conditions.** In anaerobic condition (♦), proteins functional in (*A*) nickel transport, (*B*) hydrogen metabolism, and (*C*) utilization of citrate and (*D*) gluconate were induced. Aerobic blood cultures (♢) were characterized by up-regulation of (*E, F*) heme and iron transport, (*G*) aerobic respiration and (*H*) purine metabolism. Proteins with more than 2 log_2_ difference in LFQ intensities are marked by *, while proteins with significantly increased levels but smaller differential abundance are marked by **●**. Each point shows average and standard deviation of LFQ intensities among 6 studied ExPEC strains for a specific protein.

Next, we statistically compared LFQ intensities of proteins shared between anaerobic and aerobic conditions, both for individual strains and for all collected blood cultures (supplemental Table S9). The analysis, which was based on two-sided *t* test with a false discovery rate (FDR) of 1%, identified altogether 10 and 32 proteins whose LFQ intensities increased by more than 2 log_2_ under anaerobic or aerobic conditions, respectively. A majority of these proteins showed consistent differential abundance between the two conditions in all *E. coli* strains, and only few proteins displayed LFQ intensity variations in at least two strains (supplemental Table S10). In anaerobic blood cultures we detected an up-regulation of the NikABCDE ATP-dependent nickel (II) uptake system (Fig 2*A*). Nickel acts as a cofactor for NiFe hydrogenases, key metabolic enzymes responsible for hydrogen production from glucose during *E. coli* fermentative growth (42). Correspondingly, a major subunit HycE of the formate hydrogenlyase complex had significantly increased levels in anaerobic conditions, and the quantitative profile of 9 Hyc proteins supported an up-regulation of the whole *hyc* operon (Fig 2*B*). Other proteins whose abundance increased in response to anaerobic conditions were the citrate lyase complex (Fig 2*C*) and NAD^+^-dependent succinate semialdehyde dehydrogenase Sad. Besides the proteins participating in mixed-acid and citrate fermentation, gluconate utilization was enhanced: levels of thermoresistant glucokinase GntK and both high-affinity (GntT) and low-affinity (GntU) gluconate transporters increased in response to anaerobic conditions (Fig 2*D*).

Functions of multiple proteins with significantly increased amounts in aerobic conditions were linked to iron acquisition, transport and utilization. Heme is one of the most abundant sources of invertebrate iron for pathogenic *E. coli*, and we identified six proteins linked to heme utilization and encoded by the *chu* and *dpp* operons (43, 44). Heme transporter ChuS, bifunctional peptide/heme permease DppA, and heme-binding protein ChuX showed the most significant increase in the relative amounts (Fig 2*E*). Up-regulation of the iron acquisition systems was further evident from the quantitative profile of pairs of proteins belonging to ferrous iron transporter Efe, ferric citrate transport system Fec, and the TonB-ExbBD inner membrane complex, which facilitates energy transfer to outer membrane iron transporters. In addition, proteins of two siderophore systems had increased levels in aerobic conditions (Fig 2*F*), most markedly the nonribosomal peptide synthetase EntF, which assembles the siderophore enterobactin, the enterobactin receptor FepA, the IucD protein, which participates in the synthesis of aerobactin, and the aerobactin receptor IutA.

Three protein complexes essential for aerobic respiration (cytochrome bo3 ubiquinol oxidase CyoAB, succinate dehydrogenase SdhAB, and fumarate hydratase FumAC) were significantly increased in response to the presence of O_2_ in blood cultures (Fig 2*G*). Several other enzymes involved in cellular respiration had higher abundance in aerobic conditions: citrate synthase GltA, formate dehydrogenase FdoG and respiratory nitrate reductase NarG. Finally, levels of four proteins with functions in purine metabolism (ribonucleoside-diphosphate reductase NrdA, nucleoside diphosphate kinase Ndk, and PurD and PurT purine biosynthetic enzymes) were significantly increased in aerobic conditions, and quantitative levels of proteins encoded by the *pur* operon indicated a general up-regulation of purine biosynthesis (Fig 2*H*).

##### Quantitative Analysis of Six ExPEC Proteomes Derived from Blood Cultures Reveals Minor Variations Between the Strains

**Fig. 3.**
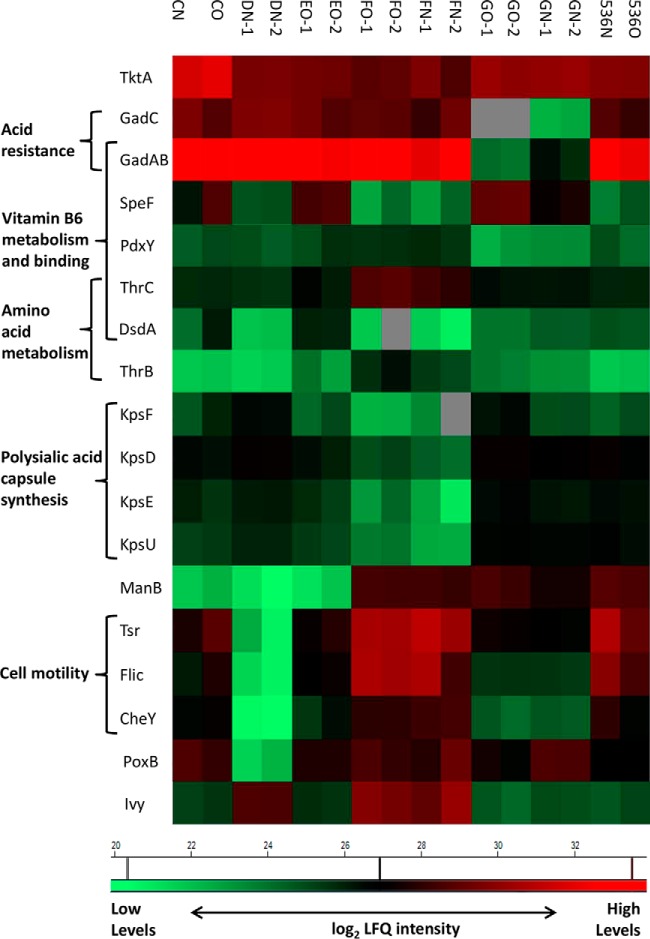
**Variations in protein expression in blood culture among ExPEC strains.** Relative abundance of 47 shared proteins between six ExPEC strains was significantly different and a selection of 18 proteins with >2 log_2_ difference in LFQ intensities is shown. These enzymes participate in metabolism of vitamin B6 and amino acids (GadABC, SpeF, PdxY, ThrBC, DsdA) and carbohydrates (TktA, ManB), polysialic acid biosynthesis (KpsDEFU) and in cell motility and virulence (Tsr, Flic, CheY, Ivy). N and O stand for anaerobic and aerobic blood culture condition, respectively. Average of log_2_ LFQ intensities is shown (minimum two replicates). Gray fields indicate missing value (protein not quantified).

We next explored differences between protein profiles of individual ExPEC strains. One-way analysis of variance (ANOVA) with permutation-based FDR correction for multiple hypotheses testing (cutoff FDR value of 1%) was used to compare LFQ intensities of 1757 proteins quantified in all studied strains. The comparison identified 47 proteins whose quantitative levels varied by more then 2 log_2_ from the respective LFQ intensity means (supplemental Table S11). Various functions in cell metabolism were represented in this group, among others enzymes participating in amino acid and carbohydrate metabolism, lipopolysaccharide biosynthesis, or protein transport. Particularly, we observed strain-specific variations in the amounts of multiple proteins involved in vitamin B6 (pyridoxine) metabolism and binding (Fig. 3). The active form of vitamin B6, coenzyme 5-phosphate pyridoxal 5′-phosphate, plays an essential role in amino acid metabolism (45), and several enzymes participating in glycine, serine and threonine metabolism showed significant differences in their relative amounts. In addition, levels of an important metabolic enzyme transketolase 1 (TktA) were significantly different among the investigated ExPEC strains. TktA provides a reversible link between glycolysis and the pentose phosphate pathway, and one of the transketolase reaction products, d-erythrose-4-phosphate, is a precursor of the pyridine ring of pyridoxine (46).

Among other significant changes was specific reduction in the amounts of proteins involved in *E. coli* acid resistance (47). The homologous glutamate decarboxylases GadA/B and glutamate antiporter GadC were detected in high levels in all strains except G5m. Further, three of the differentially expressed proteins were related to cell motility (flagellin FliC, chemotaxis protein CheY and methyl-accepting chemotaxis protein I Tsr) and their relative levels were significantly decreased in the D4n strain. We identified in total 47 proteins with various functions related to cell motility, however, only 27 were identified in the isolate D4n and 12 of these could be quantified (supplemental Table S12). At the same time, levels of two metabolic proteins, pyruvate dehydrogenase (PoxB) and phosphomannomutase (ManB), were significantly decreased in D4n. ManB had decreased amounts also in isolates C4n and E4n, which did not show any significant reduction in CheY or PoxB levels.

We detected nine protein products of the polysialic gene cluster *kps* (supplemental Table S3) that has been linked to virulence in UPEC strain 536 (48), and observed differential expression for four of them. Transport proteins KpsD and KpsE, transferase KpsU, and isomerase KpsF were detected in reduced amounts in the isolate F4d, when compared with the other strains. Another potential virulence factor that was detected as differentially expressed was inhibitor of vertebrate lysozyme Ivy (49). The levels of this inhibitor, whose role is to evade lysozyme-mediated lysis, were increased in D4n and F4d, and slightly reduced in the rest of the strains.

##### Adaptation of UPEC 536 Physiology to Three Distinct In Vitro Conditions

Flexibility of *E. coli* metabolism is one of the key factors for survival in environments with different nutritional availability (10). This important feature of ExPEC pathogenicity has however never been studied on a large scale, *i.e.* expressed proteome level. The six investigated strains behaved fairly uniformly with respect to their overall protein expression, and strain 536 could therefore be regarded as a reference for ExPEC protein expression in blood culture. We compared the blood culture-derived proteome with proteomes produced during growth of strain 536 on solid media. We found that blood and lactose agar media, which are routinely used in clinical microbiology laboratory, are relevant for our purpose as they have chemically different composition from each other, and represent contrasting culturing conditions from the blood culture.

The use of the described proteomic methodology (Fig. 1*A*), with the exception of harvesting bacterial cells in the stationary growth phase from solid media instead of a blood culture, led to the identification of 2425 and 2372 UPEC 536 proteins for the blood and lactose agar conditions, respectively (supplemental Fig. S3). ANOVA-based statistical comparison (FDR 1%) of LFQ intensities for 1,803 proteins that were quantified in all three conditions indicated significantly differential expression (>2 log_2_) for 200 proteins (supplemental Table S13). The following text describes in detail which molecular processes were altered across the conditions and the most prominent differentially expressed protein complexes (Table II).

**Table II:** Differentially expressed UPEC 536 protein complexes in response to three growth conditions

Biological function	All detected proteins within an operon	Proteins detected under specific condition (log_2_LFQ intensity*^a^*)			
		Aerobic blood culture	Anaerobic blood culture	Blood agar	Lactose agar
K^+^ transport	KdpABCDE	BDE (22.7)	BDE (22.7)	DE (24.0)	ABCDE (26.7)
Lactose utilization	LacZYAI	I (NQ)	I (NQ)	I (21.5)	ZYAI (28.7)
Thiamine biosynthesis	ThiBCDEFGHILMPQS	BCEFGHILMPQ (29.4)	BILQ (26.8)	BILMPQ (26.3)	BCDEFGHILMPQS (31.0)
Ethanolamine utilization	EutABCKLMQT	CL (NQ)	BCLMQ (NQ)	ABCKLMQT (27.3)	BCL (NQ)
Glycolate utilization	GlcABCEG	BC (26.5)	BG (26.6)	ABCEG (29.4)	BCG (27.4)
Xylose utilization	XylABF	- (NQ)	B (NQ)	ABF (24.4)	B (21.2)
N-acetylneuraminate degradation	NanAA2EKMR	AA2ER (25.3)	AA2EKMR (25.6)	AA2EKMR (27.4)	EKMR (25.7)
Fatty acid metabolism	FadABDEHIJLMR	DILMR (26.9)	DLR (26.7)	ABDEHIJLMR (30.6)	BDEHLMR (26.9)
Glycerol-3-phosphate transport	UgpABCEQ	BQ (23.7)	Q (23.12)	ABCEQ (28.8)	BCQ (25.5)
Heme and peptide transport	DppABCDF	AD (23.9)	AD (19.9)	ABCDF (30.2)	A (21.4)
Galactose transport	MglABC	AB (23.9)	AB (NQ)	ABC (30.3)	ABC (27.0)
l-arginine degradation	AstABCDE	ABD (22.3)	BD (NQ)	ABCDE (31.3)	BCD (21.3)
Hydrogen metabolism	HyaABCDEF	ABCDEF (29.4)	ABCDEF (29.3)	B (22.8)	B (NQ)
	HybABCDEFGO	ABCDEFGO (28.1)	ABCDEFGO (28.8)	BCD (23.2)	BCD (22.4)
	HycABCDEFGHI	ABCDEFGHI (29.5)	ABCDEFGHI (30.2)	EI (24.2)	BEI (23.9)
	HypABCDEF	ABDEF (28.2)	ABCDEF (27.9)	BDEF (24.9)	BDEF (23.7)
Mixed acid fermentation	FrdABCD	ABCD (30.7)	ABCD (30.7)	ABCD (28.6)	ABCD (26.6)
Nickel transport	NikABCDER	ABCDER (28.5)	ABCDER (30.6)	AER (25.8)	ABER (26.3)
Glycerol degradation	GlpABC	ABC (27.8)	ABC (28.5)	AB (21.2)	- (NQ)
Chemotaxis	CheABRWYZ, MotAB	(Che) ABRWYZ, (Mot) AB (29.2, 23.8)	(Che) ABRWYZ, (Mot) AB (30.8, 26.5)	(Che) ABRWY, - (23.7, NQ)	(Che) ABRWYZ, (Mot) AB (28.9, NQ)
Heme uptake	ChuASTUWXY	ASTWXY (29.7)	ASX (22.5)	ASTX (25.9)	ASTXY (28.2)
Polysialic acid synthesis	KpsCDEFMSTU	CDEFSTU (28.4)	CDEFSTU (28.6)	CDEFTU (27.9)	CDETU (27.1)
Transcription antitermination	CspACDEG	ACDEG (30.8)	ACEG (30.9)	CE (26.9)	ACEG (27.5)

*^a^* Average sum of quantified proteins LFQ intensities between 2 (blood cultures) or 4 (agar plates) replicates. NQ: not quantified.

##### Anaerobic Fermentative Metabolism

**Fig. 4.**
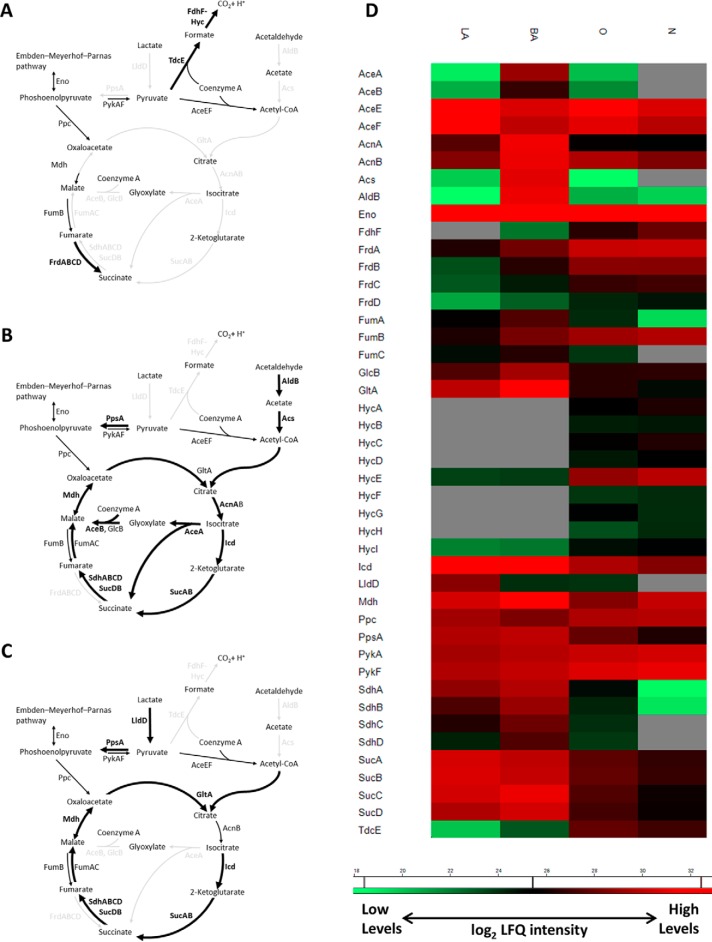
**UPEC 536 energy metabolism in response to three *in vitro* conditions.** *A*, During anaerobic growth in blood culture there were significantly increased amounts of mixed acid fermentation enzymes and formate hydrogenlyase complex. Aerobic conditions of the solid media promoted levels of multiple enzymes of the citric acid cycle and pyruvate metabolism: *B*, blood agar was characterized by up-regulation of the glyoxylate shunt, and (*C*) lactose agar by induction in lactate degradation. Proteins with more than 2 log_2_ difference in LFQ intensities when comparing conditions are shown in bold. *D*, Quantitative profiles of proteins shown on the schematic illustrations in *A*, *B,* and *C*. Average of log_2_ LFQ intensities is shown. Gray fields indicate missing value (protein not quantified). Abbreviations: O - aerobic and N - anaerobic blood culture, LA - lactose agar, BA - blood agar.

As anticipated, the energy metabolism of strain 536 was dependent on the availability of O_2_ (Fig. 4). The blood culture condition (Fig. 4*A*) was characterized by up-regulation of three membrane-bound NiFe hydrogenases, encoded by the *hya*, *hyb*, and *hyc* genes, together with their auxiliary Hyp proteins required for the hydrogenases maturation. We detected 30 Hy proteins in the blood culture samples of strain 536 but only up to 11 Hy proteins were present under the solid media conditions (Table II). That none or limited access to O_2_ in the blood cultures had significant effect on the levels of multiple proteins, was further documented by differential expression of nickel transporter (NikABCDE), fumarate dehydrogenase (FrdABCD), pyruvate formate lyase (PFL)-like enzyme (TdcE), and membrane-bound nitrate reductase (NarGHI) (supplemental Table S11).

##### Aerobic Respiration and Oxidative Stress

In contrast to the blood culture, aerobic growth of strain 536 on solid media led to a significant increase in the levels of the tricarboxylic acid (TCA) cycle enzymes (Fig 4*B*, 4*C*). It included three enzymes encoded by eight genes and cotranscribed from the *sdh* promoter (SdhABCD, SucAB, and SucCD), malate and isocitrate dehydrogenases (Mdh, Icd), and citrate synthase (GltA). Moreover, we detected an increase in levels of two aconitases (AcnA and B); both enzymes had the highest levels in response to the blood agar. The up-regulation of the AcnA expression pointed to an increase in oxidative stress during growth on blood agar (50). Accordingly, we detected induction of oxidative stress proteins SodA, Tpx, MrsBC, YgiW, and YfcG in response to both of the agar media, and SodC, MrsA, Pka, and YhbOW were particularly induced on blood agar, together with 11 proteins controlling cellular redox homeostasis (supplemental Table S13).

Growth on blood agar was unique by significant up-regulation of isocitrate lyase (AceA) and malate synthase (AceB), key enzymes of an anaplerotic pathway of the TCA cycle, the glyoxylate shunt (Fig. 4*B*). Furthermore, levels of enzymes responsible for the generation of acetyl-CoA (AldB and Acs) through dehydrogenation of acetaldehyde to acetate were induced on blood agar, when compared with the lactose agar and blood culture. Another enzyme participating in pyruvate metabolism was lactate dehydrogenase (LldD), which was exclusively up-regulated in response to the growth on lactose (Fig. 4*C*). Finally, on both solid media there were significantly increased levels of phosphoenolpyruvate synthase (PpsA) that is required for the synthesis of precursor metabolites for cellular carbon compounds through the gluconeogenesis pathway.

##### Carbohydrate Metabolism

**Fig. 5.**
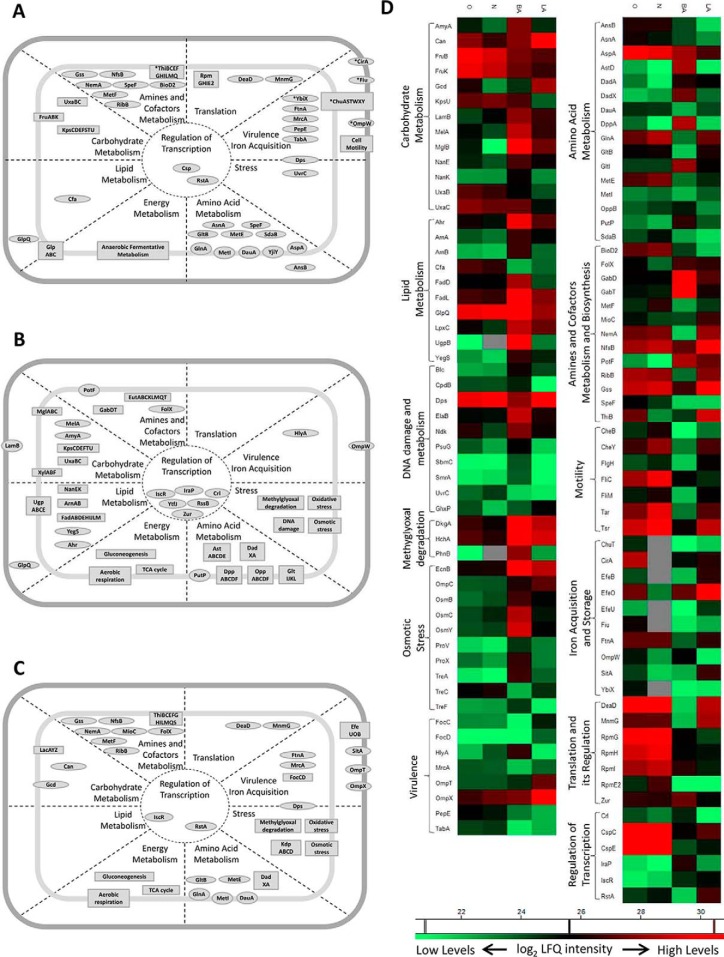
**Overview of metabolic changes for UPEC 536 in response to three *in vitro* conditions.** The model illustrates multiple proteins participating in various cellular processes, which had differential abundance in (*A*) blood culture, on (*B*) blood agar, or (*C*) lactose agar. Section (*D*) shows average log_2_ LFQ intensities of proteins with significantly differential abundances (>2 log_2_ difference in LFQ intensities) across the conditions, and functional in specific metabolic processes. Several proteins displayed higher abundance in aerobic blood culture when compared with the anaerobic condition (*). Individual proteins are shown as ovals, while rectangles indicate multiple proteins encoded by the same operon and various biological processes. Approximate localization of the proteins is indicated by their placement within the illustration of the cell.

Several co-regulated proteins appeared exclusively in samples derived from the solid media, notably enzymes required for utilization of specific carbohydrates (Fig. 5). Three proteins carrying out lactose breakdown (LacZYA) were present only in the lactose condition (Table II). In the blood culture and blood agar the genes of the *lac* operon were repressed by LacI, which was detected at low levels in all conditions. Similarly, two out of three proteins of the xylose- inducible operon (XylABF) were detected and quantified only in the blood agar condition (Table II). Increase in the relative levels in response to the cultivation on blood agar showed enzymes catalyzing hydrolysis of glucan (AmyA) and melibiose (MelA), degradation of sialic acid (NanEK), and transport of galactose (MglABC) and maltose (LamB) (Fig. 5*B*). Transport and degradation of fructose was on the other hand induced in blood culture (FruABK, EC958_3041) (Fig. 5*A*). Conditions containing blood had in common up-regulation of the *uxa* and *kps* operons, encoding enzymes for d-galacturonate degradation and polysialic acid synthesis, respectively. Conversely, on lactose agar were specifically up-regulated two metal-binding enzymes (Fig. 5*C*): carbonic anhydrase (Can) that interconverts CO_2_ and bicarbonate, and glucose dehydrogenase (Gcd), a membrane protein that catalyzes oxidation of d-glucose to gluconolactone.

##### Lipid Metabolism

Growth on blood agar showed up-regulation of liposaccharide and fatty acid metabolic processes, when compared with blood culture or lactose agar (Fig. 5). Specifically, we detected increased levels of proteins encoded by the *arn* and *fad* operons, which are involved in lipopolysaccharide modification and utilization of fatty acids, respectively. In addition, an aldehyde reductase (Ahr), a putative diacylglycerol kinase (YegS) and zinc-dependent deacetylase (LpxC), had all significantly increased levels on blood agar (Fig. 5D). The latter enzyme catalyzes the committed step of lipid A (endotoxin) biosynthesis. Interestingly, Arn proteins act in a pathway that modifies lipid A phosphates causing increased resistance to polymyxin (51).

On blood agar there were further significantly increased levels of proteins facilitating glycerophosphodiester/glycerol-3-phosphate transport (UgpABCE) and degradation (GlpQ). However, GlpQ showed high levels also in blood culture. In addition, anaerobic glycerol-3-phosphate dehydrogenase (GlpABC) was detected in all samples derived from blood cultures, but only in limited amounts in the solid media samples (Table II). Under the blood culture condition we also detected a significant increase in levels of Cfa synthase that is involved in cyclopropane fatty acid biosynthesis.

##### Amino Acid Metabolism

One of the largest variations in response to different growth conditions displayed UPEC 536 amino acid metabolism (Fig. 5, supplemental Fig. S4). On nutrition-rich blood agar the comparative proteomic analysis detected significantly increased levels of several peptide (DppABCDF, OppABCDF) and amino acid transporters (GltIJKL, PutP), whereas in blood culture there were increased levels of predicted transporter YjiY, which is induced at the onset of stationary phase in media containing peptides or amino acids (52). Levels of four enzymes participating in transport and metabolism of aspartate were increased in blood culture: periplasmic l-aspariginase II (AnsB), C4-dicarboxylic acids transporter (DauA), and two cytoplasmic enzymes catalyzing the conversion of aspartate either into arginine or fumarate (AsnA, AspA). In contrast to blood agar, where was induced uptake of periplasmic glutamate and aspartate through the Glt transporter, lactose agar and blood culture had in common significantly increased levels of cytoplasmic glutamate synthase (GltB) and gluatamine synthetase (GlnA). Similarly, transport of periplasmic succinate (DauA) increased on lactose agar and in blood culture, whereas on blood agar we identified an up-regulation of the *astCADBE* operon that encodes enzymes participating in the conversion of arginine to succinate. In the former conditions were also increased levels of enzymes responsible for methionine transport (MetI) and biosynthesis (MetE). Finally, on both solid media we observed an induction of l-alanine degradation into pyruvate (DadA, DadX), while in blood culture there were increased levels of l-serine deaminase II (SdaB), which catalyzes the deamination of serine to pyruvate.

##### Amines Metabolism

The detected proteome of strain 536 contained 8 proteins for ethanolamine utilization, and 7 of these were quantified only in the samples originating from blood agar (Table II). Further, we noticed an up-regulation of transport and utilization of putrescine on blood agar: putrescine ABC transporter - periplasmic binding protein PotF and two enzymes GabD and T, which carry out degradation of an intermediate in putrescine degradation, 4-aminobutyrate, had significantly increased levels (Fig. 5*D*). On the other hand, in blood culture we detected increased levels of ornithine decarboxylase SpeF, which catalyzes the decarboxylation of ornithine to putrescine. Metabolism of another polyamide synthesized from putrescine, spermidine, was affected by the culturing conditions: levels of bifunctional glutathionylspermidine amidase/synthetase Gss were significantly downregulated on blood agar, when compared with lactose agar and blood culture. Finally, blood culture and lactose media had in common increased levels of two proteins (NemA, NfsB) linked to the utilization of 2,4,6-trinitrotoluene (TNT) as a source of nitrogen (53).

##### Biosynthesis and Utilization of Cofactors

We detected in total 14 proteins with different functions in the biosynthesis and transport of thiamine (13 Thi proteins and IscS cysteine desulfurase) and obtained relative quantification for 12 of these. Growth on lactose agar displayed the highest levels of Thi proteins, followed by aerobic blood culture (Table II). At the same time, only 4 and 3 Thi proteins were quantified in the anaerobic blood culture and on blood agar, respectively. Also levels of proteins functional in metabolism of biotin, folate and riboflavin responded differentially to each culturing condition (Fig 5*D*). Specifically, levels of dethiobiotin synthetase BioD2 were significantly reduced on lactose agar, however, another protein involved in biotin synthesis, MioC, had highest abundance in the same condition. On blood agar we detected significantly decreased levels of folate reductase (MetF), and of 3,4-dihydroxy-2-butanone 4-phosphate synthase (RibB), a protein participating in a branch of the riboflavin biosynthetic pathway that utilizes ribulose-5-phosphate. Levels of the FolX epimerase, which is essential for tetrahydromonapterin biosynthesis, significantly increased under both solid media.

##### Cell Motility

Induction of proteins facilitating cellular motion was prominent in blood culture of UPEC 536 (Fig. 5*E*). Totally 45 proteins with various functions in cell motility were identified in blood culture, and 40 of these were described by LFQ intensities (supplemental Table S12). On lactose agar, 29 out of 41 detected proteins could be quantified, and these numbers were even lower for blood agar (30 and 15 for the detected and quantified proteins, respectively). The most significant differential abundances were displayed by four chemotaxis proteins (CheBY, Tar, Tsr) and three flagellar proteins (FlgH, FliC, FliM).

##### Iron Acquisition and Storage

Another example of adaptation to a specific growth condition was differential expression of proteins linked to iron acquisition and storage (Fig. 5E). Six proteins of the heme uptake system Chu (ChuASTUWX) had highest levels in aerobic blood culture, followed by growth on lactose agar, and the lowest expression of the Chu system was detected under blood agar and anaerobic blood culture. Similar patterns were displayed by ferric iron-catecholate outer membrane transporter CirA and catecholate siderophore receptor Fiu. On the other hand, ferrous iron transporter EfeUOB and putative iron transport protein SitA had the highest abundances on lactose agar. Outer membrane protein W (OmpW), which is also considered an iron uptake compound, had highest levels in aerobic blood culture and blood agar. Bacterial non-heme ferritin FtnA, an iron storage protein, had significantly increased levels both in blood culture and on lactose agar when compared with blood agar. Last but not least, levels of another protein participating in cellular iron ion homeostasis, a hydroxylase YbiX, were specifically increased in aerobic blood culture when compared with the solid media.

##### Transcriptional Regulation

Six transcriptional factors showed significantly different abundances in the investigated conditions (Fig. 5*E*) and seven other regulators displayed moderate changes in their relative amounts. Levels of iron-sulfur cluster regulator IscR were increased on solid media and a similar quantitative profile, although not identified as significantly different, showed the alternative sigma factor σ^S^ (RpoS) and a global regulator Fur. Two regulatory proteins that influence levels of RpoS, IraP, and Crl, together with putative regulator YtfJ implicated in stress response and protein misfolding, had the highest detected levels on blood agar. IraP is an antiadaptor protein required for stabilization of RpoS during phosphate starvation and Crl increases the activity of RpoS by direct interaction with the RpoS holoenzyme. A corresponding quantitative pattern to that of Crl and IraP was displayed by RssB, an adaptor protein that facilitates degradation of RpoS by the protease ClpXP. IraP interferes directly with RssB-mediated degradation of RpoS by interacting with RssB (54). We also noticed that the levels of protease ClpX were lowest on blood agar. RstA, a response regulator from the two-component system family and part of the PhoP/PhoQ regulon, had highest levels in the lactose condition. Finally, two proteins encoded by the *csp* operon encoding transcription antiterminators and regulators of RNA stability (cold-shock proteins) displayed significant increases in abundance in blood culture.

##### Translation

Four ribosomal proteins (RpmGHI and YkgM, a paralog of RpmE) had increased levels in blood culture, compared with the solid media. When comparing all 30S (S1-S21) and 50S (L1-L36) ribosomal proteins, we detected a certain reduction in relative quantitative levels of most ribosomal proteins on blood agar (supplemental Fig. S5). Interestingly, transcription of YkgM is repressed by the zinc uptake repressor Zur (55), whose levels were significantly increased on blood agar. Accordingly, levels of the Zur-repressed Zn^2+^ ABC transporter (ZnuABC) were lowest in the blood agar, when compared with blood culture and lactose condition. Among other proteins affecting translation and with significantly increased levels in blood culture, but also on lactose agar, were the DeaD protein, a DEAD-box RNA helicase that participates in the assembly of the large ribosomal subunit, and a conserved tRNA modifying enzyme MnmG. MnmG has been recently identified as an important regulator and determinant of bacterial virulence (56).

##### DNA Metabolism

Several differentially expressed proteins had roles in nucleotide metabolism and cellular response to DNA damage. Five members of the RpoS stress response regulon (Blc, YcgB, SbmC, YqjD, ElaB) and four enzymes functional in nucleotide and nucleic acid metabolism (YeiN, Ndk, SmrA, CpdB) had the highest detected levels on blood agar (supplemental Table S13). The highest levels of a member of UvrABC endonuclease enzyme complex (UvrC) and guanine/hypoxanthine transporter GhxP were detected in blood culture and the lactose condition, respectively. Lactose agar and blood culture had in common significantly induction of Dps, a DNA-binding protein that protects cells among others from oxidative stress and nutritional deprivation.

One source of DNA damage is exposure of cells to methylglyoxal, a toxic electrophile produced during unbalanced sugar metabolism. Degradation of methylglyoxal was induced in response to the growth on solid media and particularly on blood agar: methylglyoxal reductase (DkgA), glyoxalase III (HchA) and predicted glyoxalase (PhnB) had all significantly increased levels in the samples derived from blood agar. The activity of the Kef potassium channels limits methylglyoxal-induced DNA damage (57), and KefC and F proteins were detected at slightly increased levels on both solid media. During methylglyoxal stress, the expression of another transporter, potassium translocating Kdp-ATPase, is consistent with an enhanced K^+^ loss consequent upon activation of Kef systems (58). This high-affinity K^+^ transporter was particularly induced on lactose agar (Table II).

##### Osmotic Stress

*E. coli* grown at high osmolarity synthesize internal trehalose as an osmoprotectant (59) and we detected an induction of trehalose metabolism on blood agar; both periplasmic and cytoplasmic trehalases (TreA, TreF) that convert trehalose to glucose had significantly increased levels (Fig. 5*E*). The proteomic data further confirmed a number of proteins with osmoprotective functions whose levels increased on blood agar, and also to some degree in the lactose condition. These included five osmotically induced cell wall proteins (OsmBCEFY), four proteins that facilitate an uptake of the osmoprotectants glycine and betaine (ProVWX, OmpC), and bacteriolytic lipoprotein EcnB that is expressed in stationary phase under high osmolarity conditions from an RpoS-dependent promoter.

##### Virulence

Besides the above-described differential abundance of proteins functional in motility and iron acquisition/storage, we identified several other virulence factors whose expression levels depended on a specific growth condition (Fig. 5*E*). Accessory proteins FocCD for the F1C fimbriae had highest LFQ intensity values on lactose agar, as well as outer membrane protease OmpT and putative virulence factor OmpX involved in cell adhesion. Levels of penicillin-binding protein 1A (MrcA) were increased under the lactose condition, but also in samples derived from blood cultures. Putative virulence factors toxin-antitoxin biofilm protein TabA and peptidase E had significantly increased levels in response to blood culture and the pore-forming α-hemolysin (HlyA) had significantly increased levels on blood agar.

## DISCUSSION

Multiple characteristics contribute to extraintestinal virulence of *E. coli*, but similar or identical traits can be found in commensal and intestinal pathogenic *E. coli* strains. Previous studies showed that pathogenic and commensal *E. coli* strains can be categorized according to their metabolic capabilities (60) and that pathogenic *E. coli* use common metabolic regulators to coordinate metabolism and virulence genes expression (61, 62). It this report we explored how ExPEC adapt to different growth conditions and the prominent link between metabolism and virulence in detail. We used high-resolution MS to quantitatively describe protein profiles of five ExPEC strains purified from blood cultures associated with sepsis, and compared them with the expressed proteome of the reference UPEC strain 536. The proteomic analysis identified in total 2862 proteins, which is the largest set of *E. coli* proteins reported by shotgun proteomics to date, and an average of over 2500 proteins per strain is approaching the estimated expressed *E. coli* proteome (63).

By including both anaerobic and aerobic blood cultures for three clinical isolates and strain 536 we could investigate the influence of oxygen on the detected proteomes. Presence of oxygen had positive effects on the levels of proteins functional in aerobic respiration and purine metabolism, while no access to oxygen resulted in induction of enzymes of mixed-acid fermentation and gluconate metabolism. Stimulation of purine metabolism, which requires a significant amount of metabolic energy in the form of ATP, is not surprising under energetically more favorable aerobic conditions. On the other hand, *E. coli* utilizes gluconate through the Entner-Doudoroff (ED) pathway, which is common among obligate aerobic bacteria, but is also present in facultative anaerobes. Although the net energy yield of the ED pathway is only half of the 2 ATPs produced by glycolysis, the ED pathway has been predicted to require three- to 5-fold less enzymatic protein than glycolysis (64). The ED pathway plays an important role in *E. coli* adaptation to the intestinal milieu (65), and contributes to the pathogenicity of other enteric pathogens, *Vibrio cholera* and *Yersinia pestis* (66, 67). Our results indicate that the ED pathway is an important energy-forming pathway during ExPEC anaerobic growth on a fermentable carbohydrate and consequently might have a role in their virulence.

An evident difference between aerobic and anaerobic blood cultures was identified in the levels of proteins associated with acquisition and utilization of metal ions critical for either anaerobic (nickel) or aerobic (iron) respiration. The level of oxygen has an influence on expression of many genes that encode transporters either of ferric ion, the predominant iron form under aerobic conditions, or ferrous ion, the more abundant form in anaerobic environments (68). In our data set we detected 71 proteins related to iron uptake and transport and for most of these proteins we observed consistently higher levels under aerobic conditions (supplemental Table S12). Interestingly, this was valid not only for ferric transporters such as Fec, Fep, IutA, or Cir but also for ferrous transporters including Efe and Feo. These results help shed light on how ExPEC acquire iron under aerobic and anaerobic conditions.

Relative quantification based on LFQ scores further allowed for identification of 47 differentially expressed proteins among the six ExPEC strains, and their functional annotation included metabolism of vitamin B6, amino acids and carbohydrates, as well as cell motility and virulence. The importance of vitamin B6 in virulence of *Mycobacterium tuberculosis* and *Helicobacter pylori* has been previously described (69, 70), and suggested for avian pathogenic *E. coli* (71). Based on the strains protein profiles, isolates D4n and F4d showed a defect in cell motility and a down-regulation of polysialic acid biosynthesis, respectively. Cell motility and chemotaxis can significantly enhance the pathogenesis of UTIs caused by UPEC (16) and various types of *E. coli* infections were previously associated with differences in bacterial motility (15). Similarly, polysialic acid capsule is an essential virulence factor (17) and differences in abundance of the biosynthetic proteins might therefore influence the strains pathogenic potential. When inspecting the patient's clinical data (72), we found few differences between the patients: one showed a considerably higher inflammatory response while another was neutropenic and displayed elevated lactate, when compared with the other patients. However, no correlation between individual clinical data and corresponding *E. coli* strain protein production in blood culture could be obtained. The proteomic data nevertheless pointed out that there exists a certain degree of variation in the protein expression of individual ExPEC strains, which might have an effect on their virulence properties. Differences in the protein amounts can stem from specifics of the host environment as well as from the strain's genetic background. In conclusion, only a relatively small portion of the six strains common proteome showed alterations in protein amounts (≈2.7% of the shared quantified proteins), and likewise, only a small number of detected proteins had significantly different abundances in aerobic and anaerobic blood cultures (1.5%).

Contrary to a limited number of differentially expressed proteins among the six investigated strains purified from blood cultures, comparison of three contrasting growth conditions for UPEC 536 identified changes in over 11% of the shared and quantified proteins. Blood agar was marked by an up-regulation of the glyoxylate cycle, which is induced by growth on acetate. This finding was consistent with an up-regulation of the *fad* operon responsible for beta-oxidation of fatty acids into acetate. Another characteristic feature of UPEC 536 growth on blood agar was induction of multiple systems responsible for uptake of specific sugars, lipids, peptides and other biomolecules available on the nutrition-rich media. In a similar manner, proteins for uptake and utilization of lactose were detected in significantly higher amounts on lactose agar. The various carbohydrates derived from the solid media were probably one of the main reasons for up-regulation of proteins functional in osmotic stress response. Findings from studies describing an overlap between oxidative and osmotic stress responses (73, 74) furthermore suggest that the detected induction of oxidative stress proteins in response to the growth on solid media when compare with blood culture, was not caused entirely by aerobic growth conditions.

With a great simplification, nutrient-rich media like blood agar can be compared with the host-secreted mucus in the colon, which is a complex gel of glycolipids, glycoproteins, and a variety of sugar residues, including N-acetylglucosamine, N-acetylgalactosamine, d-galactose, fucose, sialic acids, glucuronate, galacturonate, and gluconate (75). *E. coli* growth in the intestine on nutrients acquired from mucus is well described (76), but information about how the bacterium adapts its physiology to permit survival and growth in blood is sparse. Proteins involved in nucleotide metabolism, cell wall integrity and acquisition of iron were previously described to play a critical role for growth of *E. coli* in human serum (77–79). In this study, UPEC 536 growth in blood culture appeared to be supported by several sugars (fructose and galacturonate), glycerol, and amino acids. Although the main nutrient sources of blood culture were derived from the growth additives (mainly yeast extract and peptone), the protein/peptide base of blood culture can be considered analogous to the main components of blood serum.

Amino acid metabolism was one of the most diverse across the three conditions, reflecting the distinct compositions of each medium as well as other aspects affecting the growth. For example, in blood culture aspartate was preferentially synthesized from l-asparagine by periplasmic l-aspariginase II (AnsB), however, on lactose agar we detected a marginal increase in levels of l-aspariginase I (AnsA), which carries the same reaction in the cytoplasm (supplemental Fig. S4). Up-regulation of AnsB in blood culture is consistent with the enzyme's proposed function, that is facilitating the provision of an anaerobic electron acceptor during anaerobic growth (80). Indeed, in addition to the AnsB induction, in blood culture there was an increased transport of periplasmic aspartate (DauA) and conversion of cytoplasmic aspartate to fumarate (AspA), the proposed terminal electron acceptor for anaerobic respiration.

Catabolism of another amino acid, d-serine, was documented to support bacterial growth during UTI and this abundant amino acid in human urine moreover acts as a signal for virulence gene expression (81). In this study, d-serine-responsive, positive transcription factor (DsdC) and d-serine-specific transporter (DsdX) were detected exclusively in aerobic blood cultures (supplemental Table S10), and the quantitative profile of d-serine dehydratase (DsdA) showed significant differences both between individual strains and between anaerobic and aerobic blood culture (supplemental Table S7). These results indicate that in blood culture ExPEC d-serine metabolism is dependent on oxygen level. In addition, blood culture exhibited an up-regulation of l-serine degradation when compared with the solid media (supplemental Fig. S4). Although the catalytic activity of one of the l-serine-degrading enzymes, TdcG whose levels were nonsignificantly increased (< 2log_2_ difference in LFQ intensity), is destroyed by exposure to oxygen (82), the main l-serine degradation pathway appeared to be mediated by constitutively expressed SdaB with respect to the oxygen status. Overall, ExPEC cultivated in blood culture seemed to use preferentially l-serine over d-serine as a carbon and energy source.

Iron acquisition systems are essential virulence factors for successful UPEC colonization of the urinary tract and their importance is underlined by a large redundancy (10). Aerobic blood culture and solid media had corresponding numbers of identified UPEC 536 proteins that function in iron uptake and homeostasis, and an exception was the already mentioned reduced detection of these proteins in anaerobic blood culture (supplemental Table S12). Induction of the heme uptake system Chu in blood culture suggested increased availability of heme, compared with the solid media. The function of this uptake system was proposed to be dependent on secreted hemolysin (83), nevertheless, hemolysin HlyA had the highest detected levels on blood agar indicating that expression of HlyA and the Chu system is not closely coordinated. Interestingly, a putative outer membrane iron acquisition protein OmpW whose expression is sensitive to an iron-deficient environment (84) was detected in significantly increased levels both in aerobic blood culture and on blood agar. Lactose agar was a medium with slightly acidic pH (lactose agar pH≈6.7, blood agar pH≈7.3) and was specific by up-regulation of EfeUOB. The observation was in accordance with a report showing that this ferrous iron transporter provides a growth advantage to *E. coli* K12 under aerobic, low-iron and low-pH conditions when a competing metal was provided (68). The findings on differential abundance of specific iron acquisition proteins indicate that the level of their expression is strongly influenced by environmental conditions, including tightly connected factors such as pH, available iron form and presence of other metal ions.

Planktonic growth of *E. coli* in blood culture positively affected the number of detected proteins functional in cellullar motility. The two most abundant *E. coli* methyl-accepting chemotaxis proteins, Tsr (l-serine) and Tar (aspartate/maltose), were previously identified as the main chemoreceptors responsible for mediating taxis of UPEC strain CFT073 toward urine (85), and deletion of chemotaxis protein CheW was found to attenuate UPEC in a murine model of UTI (16). Significant induction of Tsr, Tar, and Che proteins in blood culture (Table II) predicts the importance of chemotaxis also in blood. Because motility is generally important for a successful extraintestinal infection (16), expression of chemotaxis proteins can further aid in an efficient and rapid colonization of different host microenvironments.

In this study we documented that growth conditions ultimately influence levels of various proteins produced by ExPEC. Although the data described differences in ExPEC metabolism resulting from specific nutrient availability together with differential expression of multiple extraintestinal virulence traits, our findings do not directly link the changes in metabolism and individual proteins that have a role in ExPEC pathogenicity. *E. coli* as any other bacteria follows certain growth laws that put in balance energy flux and protein synthesis (86). For example, a cell growing on nutrition-rich but high osmolarity solid medium is likely to invest into the synthesis of osmoprotective proteins and at the same time reduced expression of less relevant proteins conferring motility or chemotaxis. Analogously, bacteria growing in liquid environment clearly need functional cellular motility to secure various energy sources. The presented study documented that ExPEC poses large flexibility to regulate expression of proteins needed for a particular environment. These results are therefore of importance for functional studies of extraintestinal virulence factors, as well as for deeper understanding of the pathogen metabolism under different conditions.

## Supplementary Material

Supplemental Data

## Footnotes

^1^ The abbreviations used are:

ExPEC: Extraintestinal Pathogenic *E. coli*
FDR: False Discovery Rate
HUH: Haukeland University Hospital
LFQ: Label-Free Quantification
LTQ: Linear Trap Quadrupole
MED-FASP: Multiple Enzymes for sample Digestion – Filter-Aided Sample Preparation
NCBI: National Center for Biotechnology Information
PCA: Principal Component Analysis
UniProtKB: Universal Protein KnowledgeBase
UPEC: Uropathogenic *E. coli*
UTI: Urinary Tract Infection.
