# Supplementary material for: Coordination of Metabolism and Virulence Factors Expression of Extraintestinal Pathogenic Escherichia coli Purified from Blood Cultures of Patients with Sepsis
Source: Mol Cell Proteomics. 2016 Jun 30;15(9):2890–907. doi: 10.1074/mcp.M116.060582 (PMC5013306; doi:10.1074/mcp.M116.060582)
Supplement: Supplemental Data [file supp_15_9_2890__index.html]

Coordination of Metabolism and Virulence Factors Expression of Extraintestinal Pathogenic Escherichia coli Purified from Blood Cultures of Patients with Sepsis — Coordination of Metabolism and Virulence Factors Expression of Extraintestinal Pathogenic Escherichia coli Purified from Blood Cultures of Patients with Sepsis — Extraintestinal Escherichia coli: Metabolism and Virulence — Supplemental Data 

# Coordination of Metabolism and Virulence Factors Expression of Extraintestinal Pathogenic *Escherichia coli* Purified from Blood Cultures of Patients with Sepsis

## Supplemental Data

- Supplemental\_information (.pdf, 1.0 MB) - Supplemental Figures S1-S5 Supplemental Tables S8 and S9
- Supplemental Tables (.xlsx, 44.2 MB) - Supplemental Tables S1-S13 (except S8 and S9 which are in the pdf file Supplemental Information)
