# Supplementary material for: Coordination of Metabolism and Virulence Factors Expression of Extraintestinal Pathogenic Escherichia coli Purified from Blood Cultures of Patients with Sepsis
Source: Mol Cell Proteomics. 2016 Jun 30;15(9):2890–907. doi: 10.1074/mcp.M116.060582 (PMC5013306; doi:10.1074/mcp.M116.060582)
Supplement: Supplemental Data [file 10.1074_M116.060582_mcp.M116.060582-1.pdf]

Supplementary material for:

Coordination of Metabolism and Virulence Factors Expression of Extraintestinal Pathogenic *Escherichia coli* Purified from Blood Cultures of Patients with Sepsis

Veronika Kuchařová Pettersen[1]*, Knut Anders Mosevoll[2], Paul Christoffer Lindemann[1,3], Harald G. Wiker[1]

[1]The Gade Research Group for Infection and Immunity, Department of Clinical Science, University of Bergen, N-5021 Bergen, Norway

[2]Department of Clinical Science; University of Bergen, N-5021 Bergen, Norway

[3]Department of Microbiology; Haukeland University Hospital, N-5021 Bergen, Norway

*To whom correspondence should be addressed: The Gade Research Group for Infection and Immunity, Department of Clinical Science, University of Bergen, N-5021 Bergen, Norway, Tel: +47 5597 4573, Email: veronika.kucharova@uib.no

Abbreviations

ExPEC - Extraintestinal Pathogenic *E. coli*

LFQ - Label-Free Quantification

UPEC - Uropathogenic *E. coli*

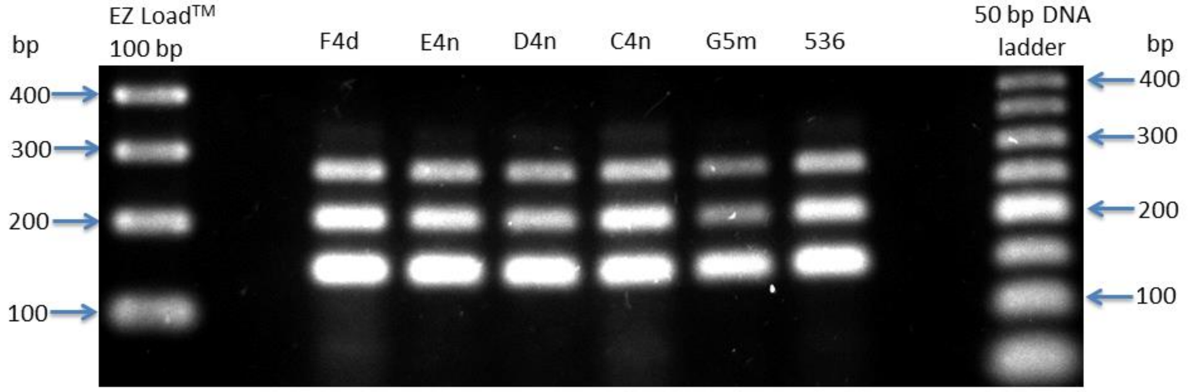

Figure S1. Phylogenetic grouping of the clinical isolates. Gel picture shows detection of *chuA* (288 bp), *yjaA* (211 bp) and TspE4.C2 (152bp). According to the Clermont phylo-typing method (21) all clinical isolates (C4n, D4n, E4n, F4d and G5m), belong to the same phylogenetic group B2 as the reference UPEC strain 536 (27)

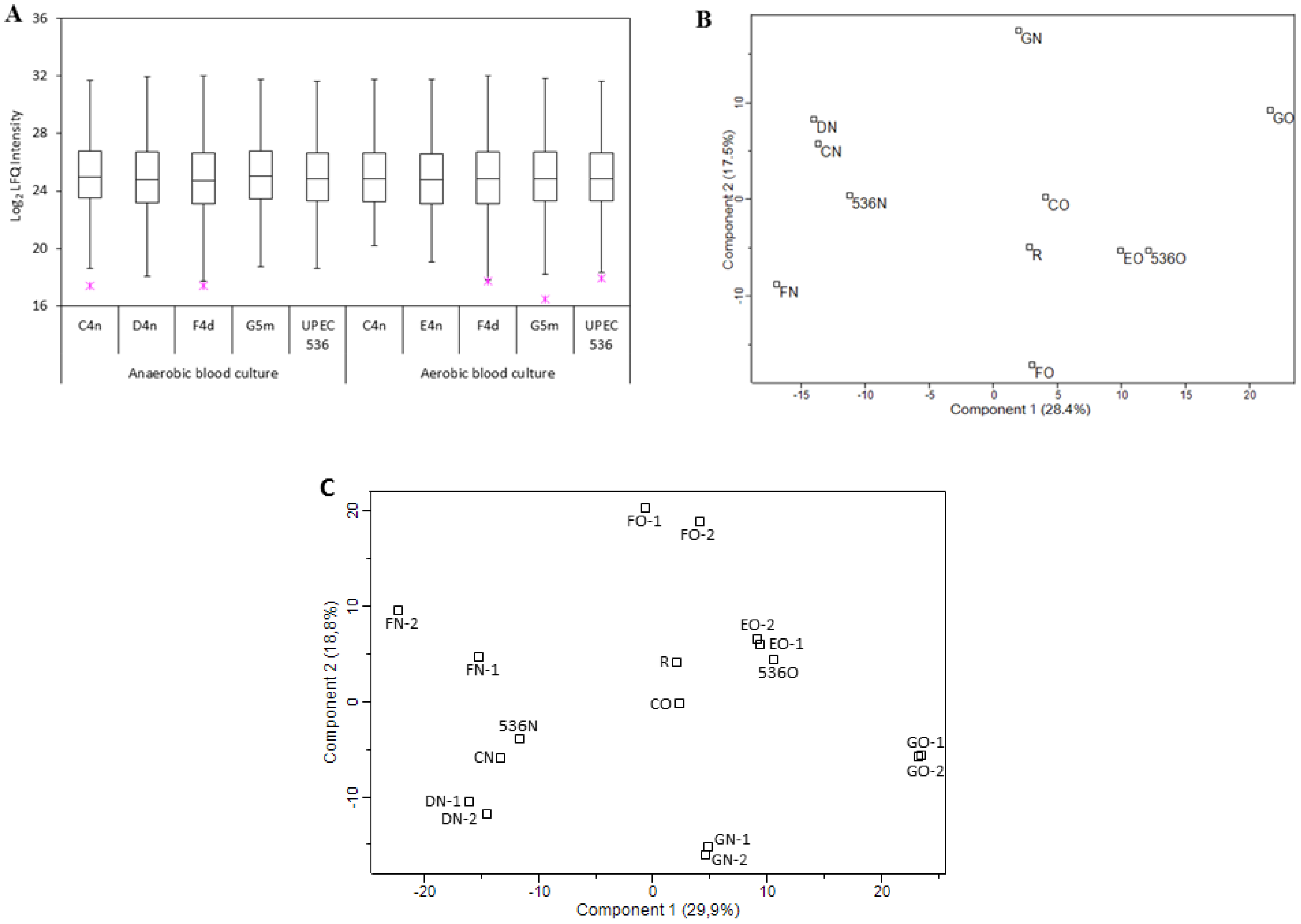

Figure S2. (A) Distribution of log_2 LFQ intensities among proteins identified for clinical ExPEC strains and reference strain 536. The medians of log_2 LFQ intensities were in the range of 24.85±0.10. Min outliers are shown as stars. (B) Principal component analysis (PCA) score plot of proteins log_2 LFQ

intensities for the individual ExPEC strains purified either from anaerobic (N) or aerobic (O) blood cultures. X- and Y-axis show the first and second principal component, accounting for 28.4 and 17.5% total variation, respectively. Control R is a mixed sample derived from anaerobic and aerobic blood cultures of five strains (C4n, D4n, E4n, G5m and 536). (C) PCA score plot of the same data set, with the distinction of individual biological replicas (*i.e.* individual blood cultures).

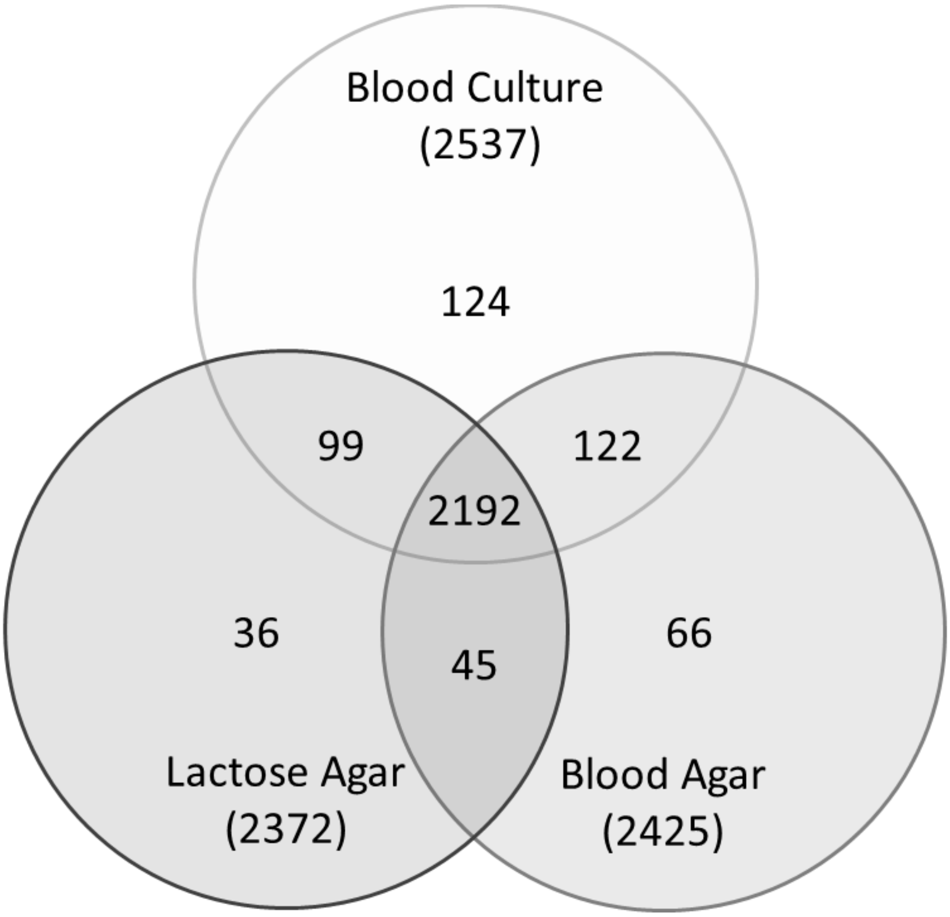

Figure S3. Venn diagram shows the numbers of UPEC 536 proteins identified in different culturing conditions.

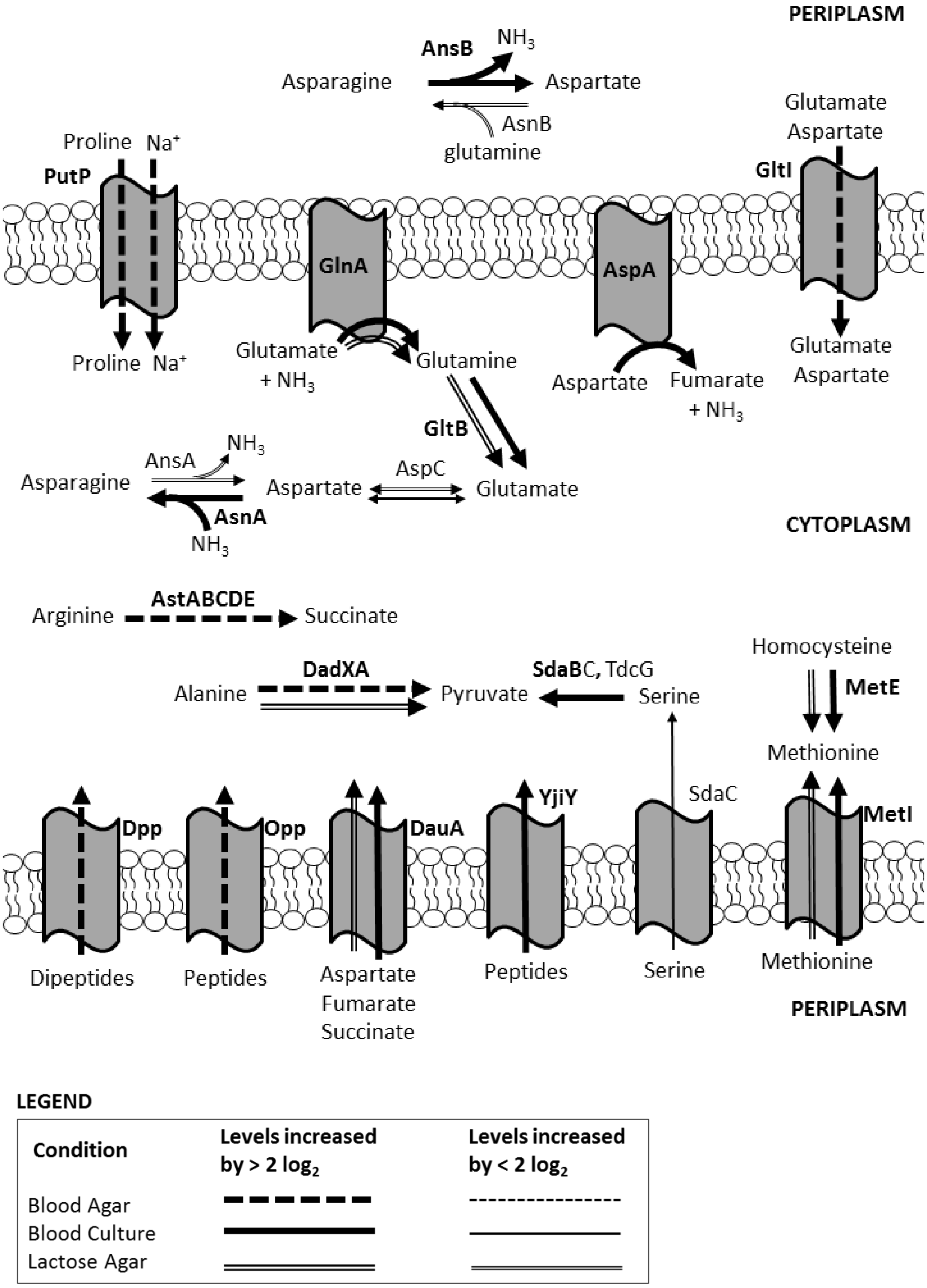

Figure S4 Reactions of amino acid metabolism catalysed by enzymes with differential expression when UPEC 536 was grown under three different culturing conditions. Proteins with more than 2 log_2 difference in LFQ intensities when comparing conditions are shown in bold.

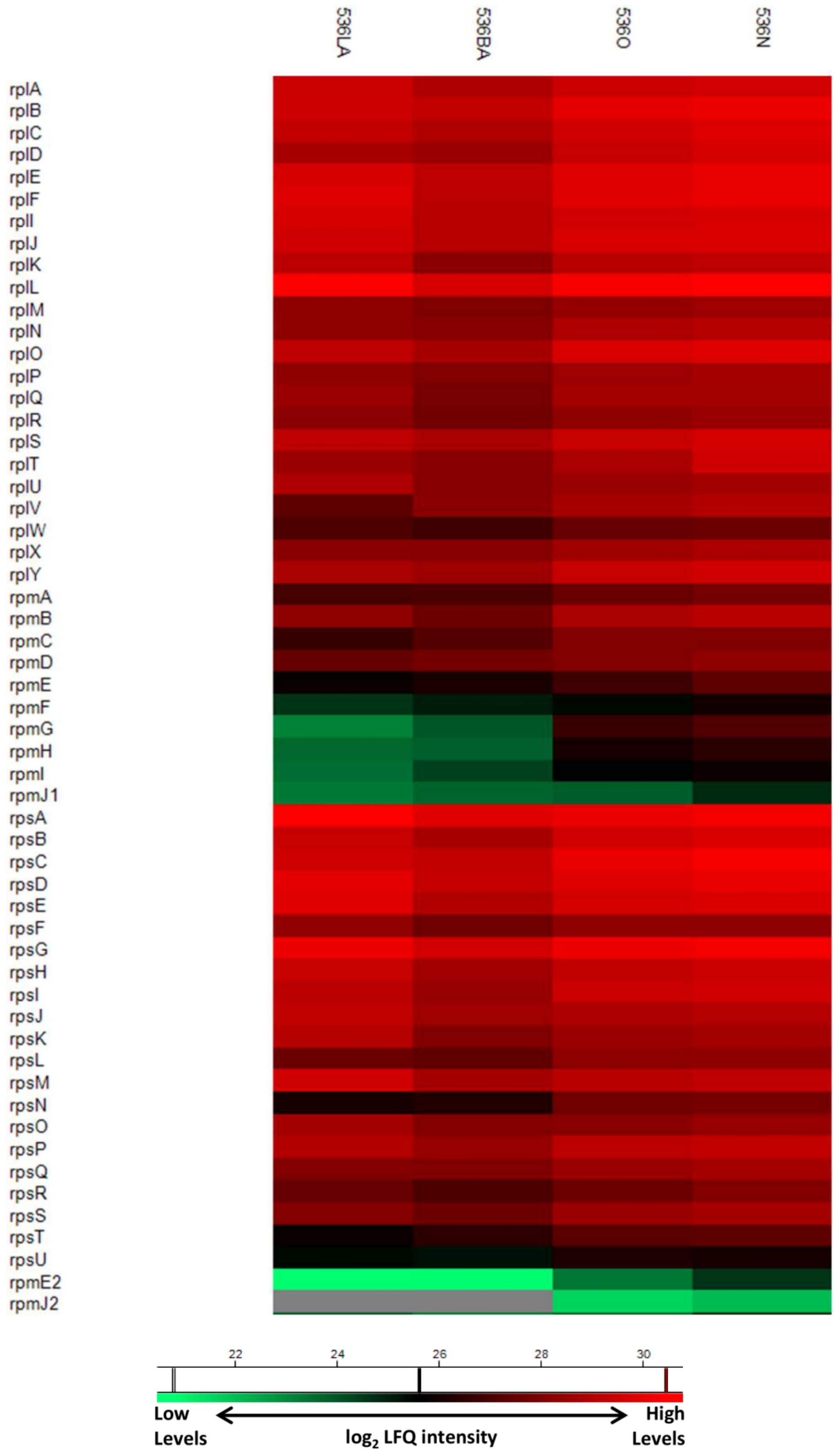

Figure S5 Quantitative profile of UPEC 536 ribosomal proteins. Abbreviations: O, N – aerobic and anaerobic blood culture, respectively, LA - lactose agar, BA - blood agar. Average of log_2 LFQ intensities is shown. Grey fields indicate missing value (protein not quantified).

Table S5. Distribution of detected proteins according to the cellular localization for six *E. coli* strains purified from blood cultures. UPEC strain 536 was in addition cultured on blood agar (BA) and lactose agar (LA).

Localization*	All strains	C4n	D4n	E4n	F4d	G5m	536	536 (BA)	536 (LA)
**Cytoplasmic**	1546	1426	1370	1435	1456	1412	1421	1377	1336
**Cytoplasmic Membrane**	615	518	491	533	540	558	526	499	493
**Periplasmic**	126	112	104	114	116	117	114	111	110
**Outer Membrane**	67	59	57	57	63	61	57	59	58
**Extracellular**	18	15	11	13	16	11	15	12	13
**Unknown**	492	399	385	401	412	387	405	367	363
**Total**	2864	2529	2418	2553	2603	2546	2538	2425	2373

* Cellular localization prediction was made by PSORTb version 3.0.2.

Table S9. Number of detected *E. coli* proteins under aerobic and anaerobic condition of clinical blood culture.

E. coli strain(s)	C4n		F4d		G5m		536		All strains[4]	
**Condition[1]**	O_1	N_1	O_2	N_2	O_2	N_2	O_1	N_1	O_7	N_7
**Proteins[2] identified (quantified)**	2448 (2037)	2310 (1844)	2491 (2124)	2469 (2100)	2390 (2047)	2395 (2008)	2424 (2092)	2346 (2009)	2833 (2509)	2788 (2420)
**Exclusive proteins**	189	51	101	78	122	127	152	74	129	40
**Common proteins (quantified)**	2259 (1801)		2390 (2012)		2268 (1928)		2272 (1939)		2763 (2380)	
**Significantly changed[3]**	1		15		8		0		29	
**Nr. of proteins with increased levels**	1	0	11	4	7	1	0	0	21	8

[1]N – anaerobic, O – aerobic. Subscript denotes number of blood cultures.

[2]Proteins detected in minimally two replicates of anaerobic or aerobic blood culture.

[3]Proteins with over 2 $\log_2$ difference in LFQ intensities associated to p-values < 0.01.

[4]All strains listed in Table 1.
